# Synthesis of Graphite Oxide with Different Surface Oxygen Contents Assisted Microwave Radiation

**DOI:** 10.3390/nano8020106

**Published:** 2018-02-13

**Authors:** Adriana Ibarra-Hernández, Alejandro Vega-Rios, Velia Osuna

**Affiliations:** 1 Centro de Investigación en Materiales Avanzados, S.C., Miguel de Cervantes No. 120, Chihuahua 31136, Chihuahua., Mexico; adriibarra26@gmail.com (A.I.-H.); alejandro.vega@cimav.edu.mx (A.V.-R.); 2 Consejo Nacional de Ciencia y Tecnología (CONACYT)-Centro de Investigación en Materiales Avanzados, S.C., Miguel de Cervantes No. 120., Chihuahua 31136, Chihuahua., Mexico

**Keywords:** graphite oxide, reduced graphene oxide, microwave radiation, modified Hummers method

## Abstract

Graphite oxide is synthesized via oxidation reaction using oxidant compounds that have lattice defects by the incorporation of unlike functional groups. Herein, we report the synthesis of the graphite oxide with diverse surface oxygen content through three (B, C, D) different modified versions of the Hummers method assisted microwave radiation compared with the conventional graphite oxide sample obtained by Hummers method (A). These methods allow not only the production of graphite oxide but also reduced graphene oxide, without undergoing chemical, thermal, or mechanical reduction steps. The values obtained of C/O ratio were ~2, 3.4, and ~8.5 for methodologies C, B, and D, respectively, indicating the presence of graphite oxide and reduced graphene oxide, according to X-ray photoelectron spectroscopy. Raman spectroscopy of method D shows the fewest structural defects compared to the other methodologies. The results obtained suggest that the permanganate ion produces reducing species during graphite oxidation. The generation of these species is attributed to a reversible reaction between the permanganate ion with π electrons, ions, and radicals produced after treatment with microwave radiation.

## 1. Introduction

Graphite oxide (GrO) and reduced graphene oxide (rGO) are interesting materials with potential applications in domains such as photonics and electronic devices, sensors, and energy storage, and as graphene synthesis precursors [1,2,3]. In general, the graphite oxidation is produced when graphite powder is exposed to strong oxidants. Different oxidation methods have been reported; Hummers and Offeman (Hummers method) developed the most important and widely applied approach for the GrO synthesis in 1958 [4]. Graphite oxidation was achieved by harsh treatment of one equal weight of graphite powders in concentrated sulfuric acid containing three equal weights of KMnO_4_ and 0.5 equal weight of NaNO_3_ [5]. Several modifications to the Hummers method have been described; for example, the Hoffmans method uses chlorate in concentrated HNO_3_ and KMnO_4_ [6], and the Brodies method employs HNO_3_ and KClO_3_ [7]; these methods have disadvantages, such as very long reaction times, and they also have dangerous reaction conditions. The analysis of the reagents’ stoichiometric quantity on the carbon material oxidation reaction is a preferred strategy for the modified Hummers method [1,2]. In all methods, oxygenated functional groups such as hydroxyl, carboxyl, carbonyl, and epoxy groups are introduced to the graphitic material, increasing the space between graphitic layers, thus decreasing the interaction between van der Waals forces [8]. Finally, the chemical or thermal reduction for the graphene structure partial recovery can also be achieved [9,10,11]. 

The microwave—assisted methods have emerged as efficient and versatile alternatives to producing varieties of carbon materials such as graphene sheets, graphene oxide (GO), or rGO, presenting advantages such as energy transfer instead of heat transfer, quick and volumetric heating, eco-friendliness, and higher safety; they can increase reaction rates by orders of magnitude compared with traditional heating [12,13,14,15]. Another advantage of microwave radiation (MWR) is the heating of the reaction mixture uniformly and rapidly, due to the difference in the reactant and solvent dielectric constants [16].

One of the strategies used for graphene or rGO synthesis is the thermal reduction through MWR starting from GrO as a precursor material [17]. Recently, a microwave-assisted rapid method was reported for the GrO reduction in metal-organic frameworks derived ZnO suspensions for photocatalytic applications [18]. Kim et al. reported the preparation of rGO/Nickel cobalt double hydroxides composites by a one-pot microwave-assisted synthesis. During the process, metal ions formed nickel and cobalt hydroxides and simultaneously GrO is reduced to rGO [19]. A theoretical analysis of chemical transformation occurring in GrO upon microwave irradiation was reported by Vitaly. They showed that microwave heating leads to a fast temperature increase, facilitating the rapid removal of most oxygen-containing functional groups on timescales that are too short [20]. The method reported for Hassan et al. allows the rapid chemical reduction of GrO using a variety of reducing agents in either organic or aqueous media [16]. However, the main use of MWR is in the synthesis reactions of organic, inorganic, and polymer compounds. The novelty of our strategy in this work, i.e., to establish a methodology and the respective study of the MWR used on the Hummers method with a graphite precursor, and not directly from GrO as it has been previously reported. 

**Figure 1:**
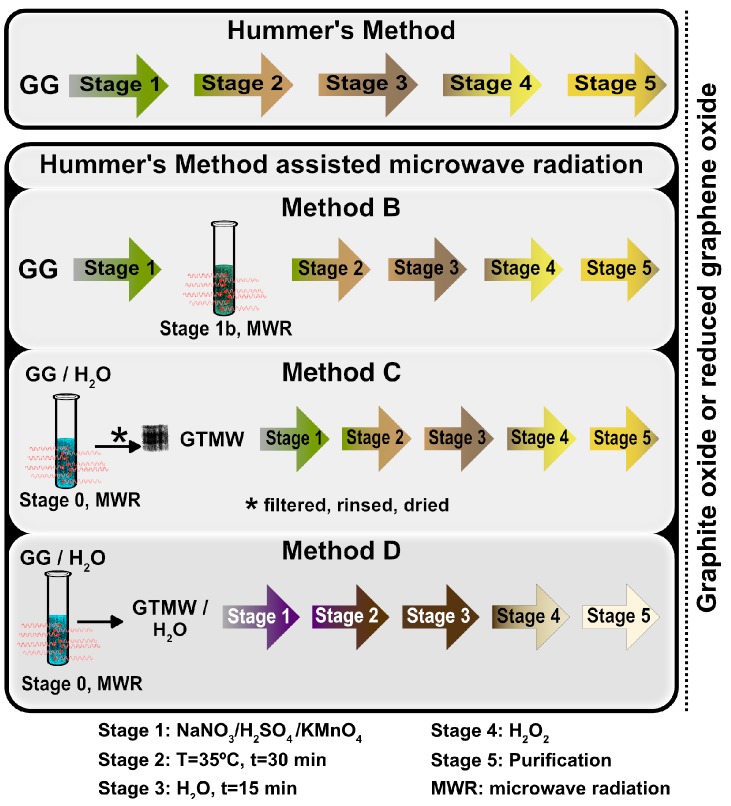
Schema of reactions of the different methodologies.

Herein, we are studying the GrO synthesis with diverse surface oxygen content through three (B, C, D) different modified versions of the Hummers method-assisted MWR compared with the conventional graphite oxide (cGO) sample obtained by Hummers method (A). The first methodology, named A, is the Hummers method composed of 5 stages, as described in the experimental part. The second methodology, named B, is characterized by applying MWR on stage 1b (Figure 1). The third and fourth are similar methodologies, in which ground graphite (GG) dispersed in water was treated with MWR (stage 0) before performing the oxidation reaction, named C and D, respectively. Nevertheless, in the methodology C, the GG dispersed in water treated with MWR (GTMW) is rinsed, filtered, and dried previous to the oxidation reaction. Finally, the characterization of different materials was carried out by X-ray diffraction (XRD), X-ray photoelectron spectroscopy (XPS), Fourier–transform infrared spectroscopy (FTIR), Raman spectroscopy, Field–emission scanning electron microscopy (FESEM), and field–emission transmission electron microscopy (FETEM). 

## 2. Results and Discussion

The GrO samples with diverse oxygen content were synthesized by three different methodologies using Hummers method-assisted microwave radiation (Figure 1). These products were compared with the cGO studying the chemical composition and structure. The well-known synthesized material by the Hummers method is called cGO. The samples of different experiments were named according to the methodology, i.e., B1, B2, B3 (methodology B); C1, C2 (methodology C); and D1, D2 (methodology D). All the methodologies were based on the same type of GG with an average particle size of 4 μm (Figure S1). The microwave reactor (Anton Paar, Graz, Austria) conditions were a power of 900 W and a frequency of 2455 MHz for all experiments. The characterization of GG, GTMW, and other experiments are present in the supplementary material. 

### 2.1. X-rays Diffraction

**Figure 2:**
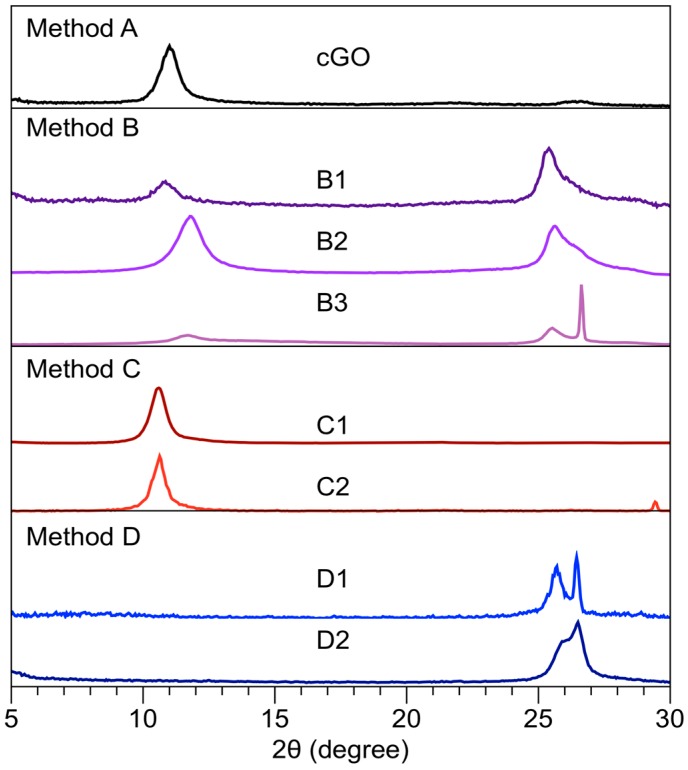
X-ray diffraction pattern of method A (cGO), method B (B1, B2, B3), method C (C1, C2), and method D (D1, D2).

The XRD (Bruker, Karlsruhe, Germany) characterization (Figure 2) of the GrO samples synthesized under the different methods is shown in Figure 2. The diffraction pattern corresponding to cGO (Figure 2) presents a peak corresponding to 11.0°, in accordance with previously reported values [2]. The results of the diffraction patterns and interplanar distance of all the synthesis methods are presented in Table S1; in some cases, a deconvolution in the range 24–27 (Figure S2) was necessary.

The diffraction patterns of all methodology B samples are different to cGO. The diffraction pattern of sample B1 (MWR conditions *t* = 5 min, *T* = 60 °C) shows a peak at 10.8° (8.2 A) and another at 25.3° (3.6 A) corresponding to planes 100 and 002, respectively. Similar behavior is observed in sample B2, which presents an exposure time of 20 min and a temperature of 60 °C. Nevertheless, in sample B3, MWR conditions *t* = 20 min and *T* = 80 °C, a third peak is shown on the diffraction pattern at 26.6 corresponding to graphite. The carbon material “partially oxidized” suggested that the MWR has a similar effect like in the chemical reduction of GrO [21,22]. The XRD peaks associated with the GrO reduction chemistry are in a range of 24.0 to 25.5 [23,24,25,26].

The GrO synthesis mechanism has been widely discussed, suggesting the oxidized structure formation during Hummers method final stages [27,28]. However, the diffraction patterns of the methodology B are similar when GrO is synthesized with a low amount of potassium permanganate (2–3 g) through the Hummers method, according to the results published by Krishnamoorthy et al. [2]. Figure S3 shows the diffraction patterns of graphite from stage 1 prior to MWR compared to the three samples obtained according to methodology B. The comparison of the diffraction patterns before the MWR and the end stage of methodology B reveal a slight to moderate increase in the peak corresponding to the 100 plane.

The graphite and water treated with MWR can generate delocalized π–electrons and dissociation in radicals and ions, respectively. Menéndez et al. [29] reported that the graphite materials can reflect a considerable fraction of MWR, increasing the kinetic energy of the delocalized π–electrons that are promoted to jump out of the material, resulting in the ionization of the surrounding atmosphere. The additional substance that is susceptible to dissociation by MWR is the water molecule that presents higher mobility of solvated electrons in an applied electromagnetic field; moreover, it contributes to microwave energy absorption, in accordance with Vaks et al. [30]. The irradiated water is decomposed in radicals or ions such as ·OH, OH^−^, H^+^, and H·, mainly. Also, trace amounts of hydrogen peroxide can be formed from two ·OH radicals [30]. Specifically, in methodology B, MWR was applied at stage 1b (Figure 1), in which the sulfuric acid used in the experiments presents 3.8% water, suggesting the formation of ions, radicals, and π–electrons with an effect on the oxidation reaction. 

The methodologies C and D were achieved with the purpose of studying the effect of delocalized π-electrons and water content on the oxidation reaction observed in the methodology B. Therefore, the material was filtered, rinsed, and dried in the methodology C. In the experiments from the methodology D, the material was placed with all the solvent, and it contained the delocalized π-electrons and/or radical. Different experiments were carried out to verify information mentioned above, in particular at stage 0 (Figure 1) of methodology C and D. The experiments consisted of measuring the physicochemical properties of the dispersion GG in water before and after MWR (conditions *t* = 20 min, *T* = 80 °C). The pH and electrical conductivity were measured at room temperature before and after the treatment by MWR. Other analyzed graphites were natural graphite powder (GN), GN-treated MWR (GNT) and natural graphite flakes (GF), and GF-treated MWR (GFT). The water used for the experiments has an electrical conductivity of 9 μS and a pH of 7. Graphites (GG, GN, and GF) have an electrical conductivity and pH in the dispersion with the water of 9 μS and 7, for each one. After the treatment with MWR, the electrical conductivity increases in all cases, registering values of 45, 53, and 57 μS, for GTMW, GNT, and GFT, respectively, see Table S2. These experiments were performed using the same amounts of water and graphite, varying only the particle sizes of the latter, without the addition of another reagent.

Another theory was the thermal reduction via MWR. However, the conditions reported in the literature present extended periods of times (4–7 h), a temperature at 100 °C, the use of reducing agents [31], thermal annealing at 300 °C under argon before exposure to microwaves [8]. Furthermore, a theoretical study of the chemical transformations occurring in GrO through MWR at high temperatures showed the removal of an over 90% of oxygen groups by MWR without destroying the graphene sheet [20]. The GTMW diffraction pattern does not present a change in the base, or the peak shifted at 26.5° to suggest a thermal exfoliation (see Figure S4). In conclusion, based on the temperature and time periods, we discard a thermal reduction via microwave. 

Therefore, we can conclude that it is possible to establish the generation of electrons, ions, and radicals from the MWR on the dispersion of graphite in water, thus reducing permanganate ions and also producing other states or species of manganese during oxidation reaction with an effect on graphite oxidation. However, the oxidations of organic compounds by potassium permanganate are usually multi–stage processes; specifically, the graphite oxidation is developed with the ion permanganate (VII) [27]. The reduction in the permanganate ion to an oxidation manganate state has been reported in alkaline pH aqueous systems where it is stable [32], and it is considered to be responsible for an alkene oxidation reaction decrease [33]. The olefins oxidation through manganate has been reported with slow reaction speed and poor performance when not diluted enough [34]. The oxidizing powers of the MnO_4_ anions decreased remarkedly in the order of (MnO_4_)^−^ > (MnO_4_)^2−^ > (MnO_4_)^3−^ [35]. The reaction between potassium permanganate and sulfuric acid produces permanganyl ion (MnO_3_)^+^ (Equation (1)) in the Hummers method [36,37,38].

KMnO_4_ + 3H_2_SO_4_ → K^+^ + MnO_3_^+^ + H_3_O^+^ + 3HSO_4_^−^ (1)

The results of the XRD patterns show that it is possible to obtain GrO under the conditions of the methodology C synthesis. Figure 2 shows the XRD patterns of the samples C1 and C2 with an exposure time of 5 min and 20 min, respectively, prior to Hummers method. The comparison of the methodology C diffraction patterns with the cGO sample shows the same behavior, establishing that there is not an MWR effect on the oxidation reaction due to the removal of the water and delocalized π–electrons through rinsing. Nevertheless, when synthesizing without removing the solvent and delocalized π–electrons (methodology D), the behavior is different in both cases; a broad peak can be observed in a range of 25–28 of XRD pattern (Figure 2), suggesting the rGO formation compared with cGO. On the other hand, Figure S5 shows the diffraction patterns of the D2a and D2b experiments, analyzing the effect of untreated and treated water with MWR on oxidation reaction using GG. The peaks are shown at 26.3 and 25.8 with an FWHM of 1 and 0.95, and an increase in their base is observed in D2a and D2b experiments, respectively, thus establishing that the treated water has an important role on oxidation reaction and it also has greater relevance than the delocalized π–electrons. Finally, the D2 diffraction pattern is the result of the sum of the patterns D2a and D2b, confirming the degradation of permanganate ion and therefore a decrease in the material oxidation. Based on the XRD results, it can be established that the addition of water to the system (Method D) causes the degradation of the permanganate ion caused by the ionization of the water producing manganate ion (Equation (2)). However, in the absence of OH radical, it does not allow the reaction to be reversible. If the water is treated with MWR, it allows the reaction to be reversible when decreasing the oxidation reaction rate.

(MnO_4_)^−^ + (OH)^−^ ⇌ (MnO_4_)^2−^ + (·OH) (2)

The manganate ion (MnO_4_)^2−^ is quite stable in alkaline solution, and the green coloration of the reaction solution during many oxidations by alkaline permanganate is evidence of its presence; however, the reaction system is under acidic conditions because of its rapid disproportionation; therefore, it is unstable in its chemical equilibrium. Other reactions that are possible through electron transfer are described in Equation (3), reported by Symons [39] and in Equation (4).

4(MnO_4_)^−^ + 4(OH)^−^ ⇌ 4(MnO_4_)^2−^ + 2H_2_O + O_2_ (3)

(4) $${\left({\mathrm{MnO}}_{4}\right)}^{-}+e_{\mathrm{graphite}}^{-}\to {\left({\mathrm{MnO}}_{4}\right)}^{2-}$$

### 2.2. XPS and FTIR Spectroscopy

**Figure 3:**
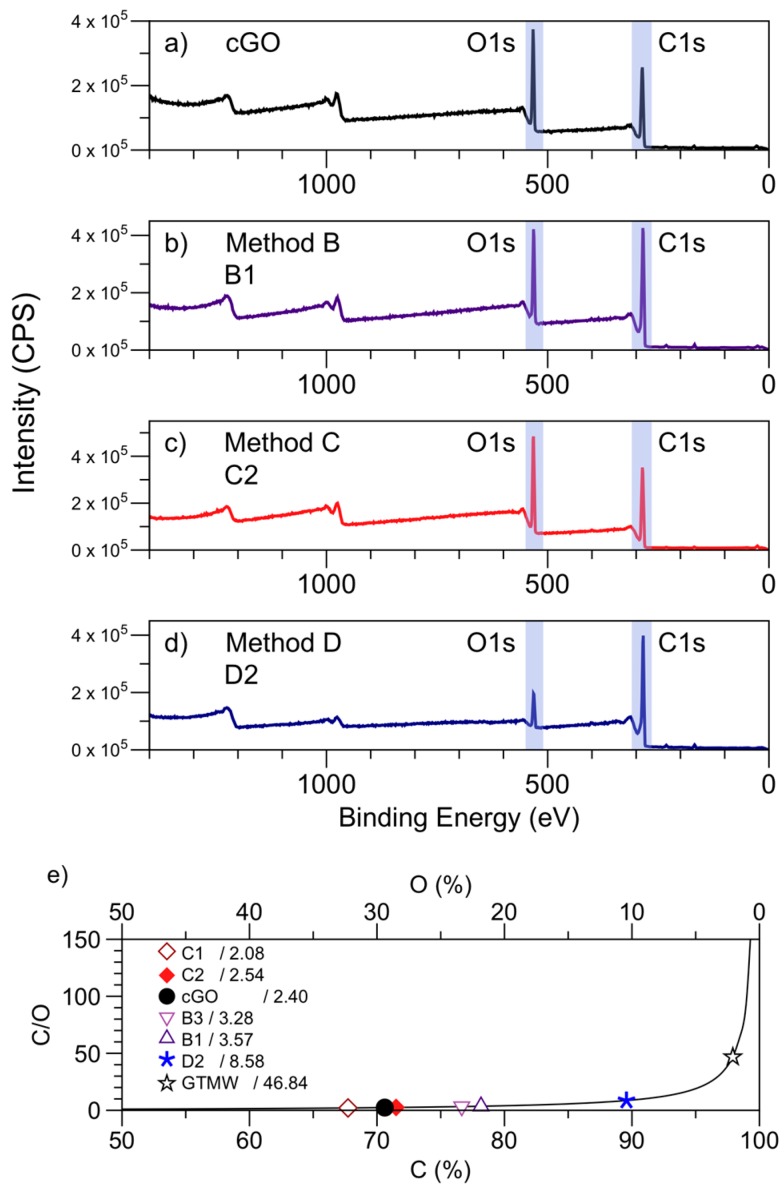
(**a**–**d**) XPS survey spectrum of each of the methods and (**e**) ratios C/O (surface).

Further information about the surface oxygen content was analyzed by XPS (Thermo Scientific, Paisley, UK) and functional groups through FTIR (PerkinElmer, Waltham, MA, USA). Figure 3a shows a typical XPS survey spectrum of each method. The peaks observed at 284.8 eV and 532.8 eV were originated by excited photoelectrons from the C1s and O1s levels, respectively [40].

The results determine that only C and O significantly contribute to the GrO chemistry surface. The feature in the binding energy ranges from 960 to 1030 eV, and it is attributable to the carbon KVV Auger transitions. Figure 3e, the values of the C/O ratio (surface) of all the methods are shown. The samples synthesized by the method C (Figure 3c) showed the highest amount of atomic oxygen content, just like cGO (Figure 3a), with a MWR time from 5 min (C1) to 20 min (C2) previous oxidation reaction time, establishing that the removal of dissociated water and delocalized π-electrons through rinsed graphite proceeds with the oxidation reaction. The synthesized samples under the method B (Figure 3b) conditions with values in the order of 3.4 suffered a slight atomic oxygen content decrease compared with cGO (Figure 3a). The low water content present in sulfuric acid and MWR at stage 1b reduces ions, radicals, and the number of delocalized π-electrons; for these reasons, the surface oxygen content decremented. Finally, rGO was obtained from the sample D2 (Figure 3d, Method D); a decrease in the peak O1s compared with cGO (Figure 3a) can be clearly observed, establishing that the graphite dispersed in water treated with MWR has an effect on oxidation reaction. Previous studies reported rGO with C/O ratios of 4.5 [41], 2.75 [21], 6.23, 10.5, and 11.6 [42]; in all cases, there is an increase in the C/O ratio when the chemical reduction through MWR takes place regarding the oxidized material. Figure S6 shows XPS survey spectrum of GG and GTMW with an atomic oxygen content of 2% and ratio C1s/O1s of 46.84 for both cases. In summary, there is not an increase in atomic oxygen content in the sample GTMW after MWR under the conditions *t* = 20 min and *T* = 80 °C.

**Figure 4:**
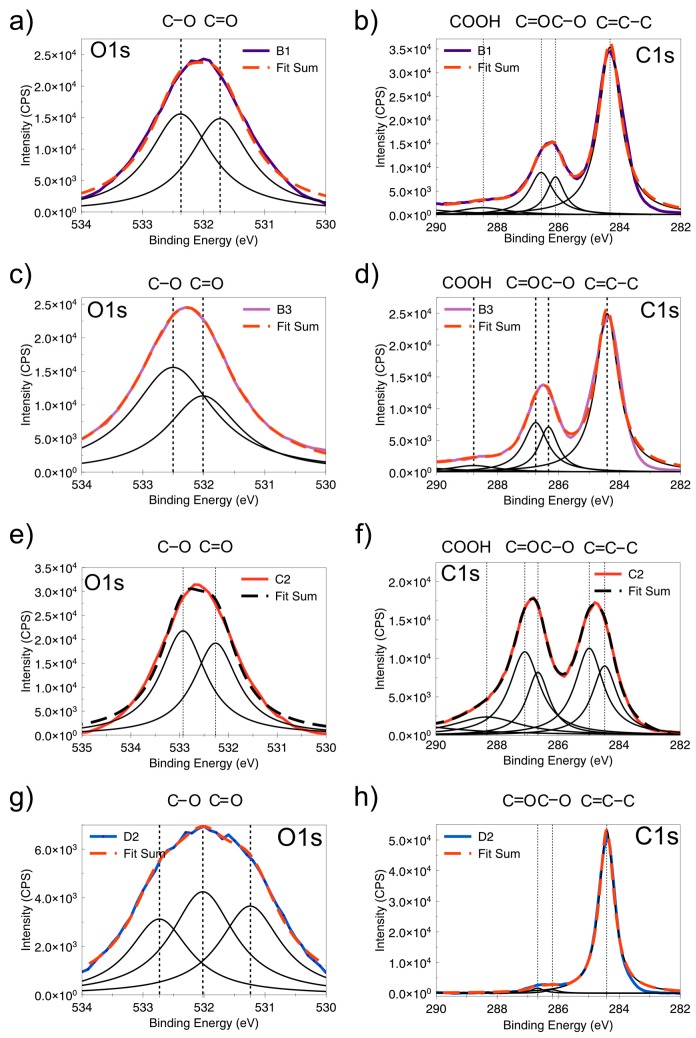
Deconvolution of the C1s and O1s peaks. (**a**,**b**) B1, (**c**,**d**) B3, (**e**,**f**) C2, and (**g**,**h**) D2.

The deconvolution of the C1s and O1s peaks of mentioned methods shown in Figure 4. Therefore, the C1s peaks were fitted with 5 peaks: sp^2^ (284.6 eV), sp^3^ (285.2 eV), C–O (286.8 eV), C=O (288.9 eV), and π-π* shake-up feature (291.0 eV). The O1s spectra from samples B1, B2 (method B), C2 (method C), and D2 (method D) could be deconvoluted into two peaks at 583, 583.6 eV, which are associated with C–O and C=O groups, respectively [43].

**Figure 5:**
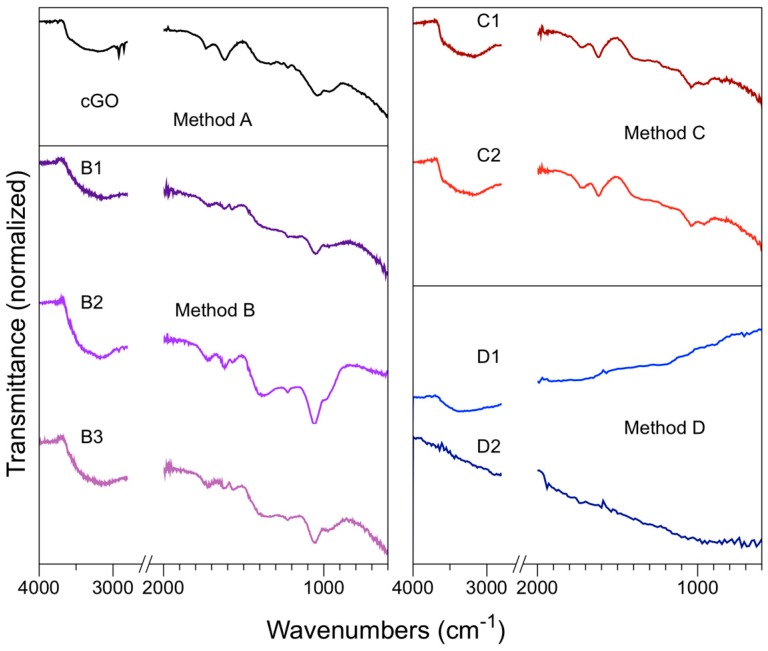
FTIR spectrum of method A (cGO), method B (B1, B2, B3), method C (C1, C2), and method D (D1, D2).

Finally, FTIR was used as a qualitative characterization for the identification of the functional groups of different materials obtained from these methods. Figure 5 shows infrared spectra of the cGO, B, C, and D methods, presenting the typical vibrations according to what it is reported [44].

The samples B1, B2, and B3 (method B) infrared spectra (see Figure 5) present a broad band in the region of 3000 cm^−1^ at 3700 cm^−1^ that it is attributed to stretching vibrations of the hydroxyl group (O–H) characteristic of the bond present in the carboxylic acids. This signal can also be related to the vibration of the residual water bond (O–H) trapped between the GrO sheets [45]. In the sample B2, two absorption peaks are observed in the region of 2920 cm^−1^ and 2848 cm^−1^, corresponding to symmetric and asymmetric vibrations of the C–H bond. An absorption band at 1381 cm^−1^ and 1342 cm^−1^ in the samples B2 and B3 appears, respectively. This band is related to the flexion of hydroxyl groups (C–OH) on the basal plane of GrO [46]. There is a characteristic absorption band between 1620 cm^−1^ and 1626 cm^−1^, corresponding to the double bond stretching vibrations C=C located at the edges of the graphene oxide [47].

In the region of 1726 cm^−1^, characteristic absorption peaks are observed from the carbonyl group C=O stretching present in carboxylic acids. This type of functional group is present at the end of the GrO layers [48]. The oxidized samples showed absorption bands in the region of 1053 to 1061 cm^−1^ and 1223 to 1225 cm^−1^, corresponding to the C–O–C bond stretching vibrations, which are a characteristic of the epoxy functional groups.

The stretching vibrations of the C–O bond located around 1225 cm^−1^ and 1381 cm^−1^ corresponding to carboxylic acids can overlap with the hydroxyl group (C–OH) flexion. This signal indicates that a change in hybridization of the sp^2^ to sp^3^ carbon has been achieved [49]. The method C samples show the same vibrations bands as in methodology B and cGO. Only methodology D presents differences with respect to the samples of the other methods due to the low oxygen content and, therefore, functional groups; it has more in common with GG and GTMW (see Figure S7).

### 2.3. Raman Spectroscopy

**Figure 6:**
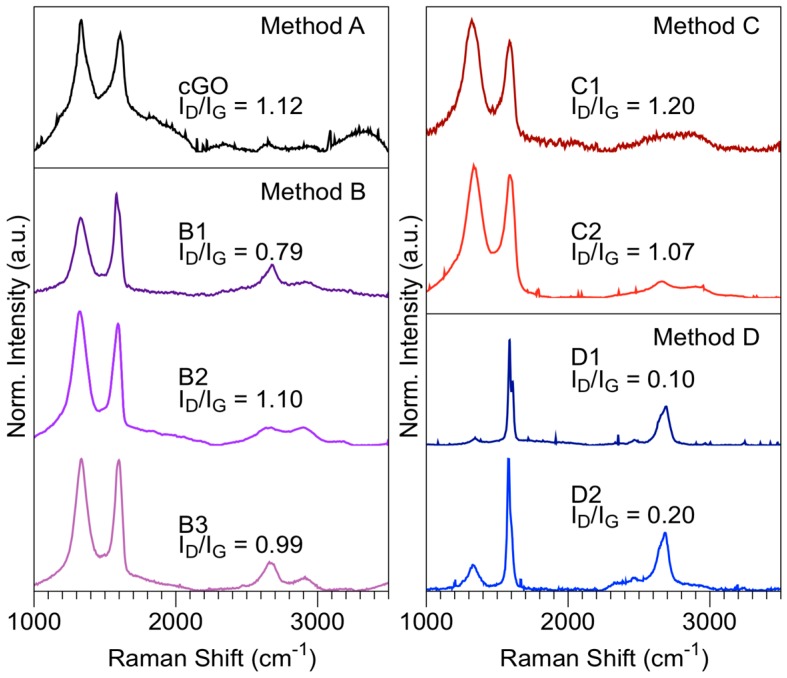
Raman spectra of method A (cGO), method B (B1, B2, B3), method C (C1, C2), and method D (D1, D2).

Raman (HoribaJobin Yvon, Longjumeau, France) spectroscopy is a powerful tool for examining the structural characterization of graphitic materials. Figure 6 shows the Raman spectra obtained by each of the methodologies. The level of disorder in the graphene was determined through the peak ratio intensities *I*_D_*/I*_G_. The methodology B presents a peak ratio intensities D and G slightly lower or equal than the value cGO, when it is irradiated under the following conditions: *t* = 5 min; *T* = 60 °C, or *t* = 20 min; *T* = 60–80 °C, respectively; suggesting that there is an effect of MWR on the methodology B, specifically at stage 1b. These results suggest that there is an oxygen atoms reduction in the graphite during the oxidation process, and consequently, a mixture of layers with or without oxygen content, as suggested by the characterization XRD. The graphene layers suffer from cracking ascribed to thermal decomposition when the radiation microwave exposure time is over 20 min and the temperatures are higher than 80 °C, denoting that the layer surface oxygen content is similar to method B. This effect has also been reported in the mechanical exfoliation [50]. The *I*_D_*/I*_G_ value slightly decreases from 1.20 for sample C1 to 1.07 for sample C2 when the MWR exposure time increased. Methodology C presents behavior similar to that obtained for cGO, exhibiting a high number of defects in the graphene layer caused by oxidation (sp^2^) carbon. Method D shows the fewest structural defects due to permanganate ion chemical reduction. The Raman spectrum deconvolution of the D1 and D2 samples in the range 1200–1800 cm^−1^ shows a main peak at 1584 cm^−1^ and 1578 cm^−1^, respectively, indicating the presence of graphite few layers [51]. There are also peaks ~1600 cm^−1^ associated with GrO [52]. 

The number of layers was determined based on the deconvolution of the band 2D, peak ratio intensities, *I*_2D_*/I*_G_, as well as the position and shape of these peaks. The analysis was performed only for the experiments B1, D1, and D2 because it presented a value of lower defects (*I*_D_*/I*_G_*)* in reference to the other materials (see Figure 6). Table S3 shows the peak ratio intensities of *I*_D_*/I*_G_ and *I*_2D_*/I*_G_. The samples B1, D1, and D2 present an *I*_2D_*/I*_G_ of 0.34, 0.37 and 0.42, respectively. Figure S8 shows the Raman spectra deconvolution, band 2D, from samples B1, D1, and D2. Method B (sample B1) presents a single peak at 2673 cm^−1^ (Figure S8) in the region 2600–2700 cm^−1^, as well as the peak associated with a chemical oxidation 2919 cm^−1^ [52]. The peak 2D deconvolution of the sample D1 reveals three bands at 2641 cm^−1^, 2664 cm^−1^, and 2693 cm^−1^; in contrast to the experiment D2 presents peaks at 2646 cm^−1^ and 2687 cm^−1^ (Figure S8). The analysis of the 2D peak shape from the experiments D suggests layers greater than or equal to 5, while the sample B1 is similar to the bulk graphite, according to the results reported by Ferrari et al. [53]. Besides, the FWHM is of 60 cm^−1^ and 76 cm^−1^ to D1 and D2, respectively [54]. The deconvolution band around 2645 cm^−1^ in both experiments of method D suggests a minority population of single-layer graphene (SLG). This method can be optimized through an exfoliation via ultrasound or thermal energy, or both, with the purpose of obtaining a greater quantity of SLG [55,56]. 

### 2.4. Electron Microscopy

**Figure 7:**
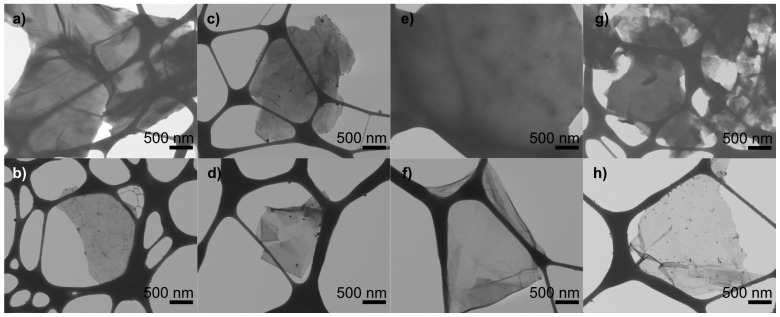
FESEM micrographs of the different GrO samples. (**a**) cGO, (**b**) B1, (**c**) B2, (**d**) B3, (**e**) C1, (**f**) C2, (**g**) D1, and (**h**) D2.

The materials were also characterized by FESEM (JEOL, Akishima, Japan) to observe their oxidation or damage compared with the electron micrographs of the Hummers method. In general, different levels of stacked material are produced by the methods described above, and according to the obtained results we would expect to see partial and total damage in the behavior of rGO for the methods B, C, and D, respectively. The sample of cGO presents a leaf-shaped morphology, with several layers (Figure 7a). It was also detected that there is damage in the graphite structure when observing the areas with greater agglomeration that are shown in darker tone, as well as a high wrinkling rate as a result of the oxidation process [57,58].

The samples B1, B2, and B3 (methodology B) present laminar morphology (see Figure 7b–d), unlike the cGO, in which a degree of greater delamination and layers with greater transparency have been obtained in these samples, which are translucent to the copper membranes that contain the samples of the method B. It is important to mention that the sheets obtained do not present wrinkling or significant folds in their structure, which is a common problem present in this type of synthesis. The only sample that showed slight folds at the edges of the sheet was B3 (Figure 7d). The results of this methodology produced homogeneous and few layers of opaque to transparent graphene oxide with sheet size between 1.2 and 3.1 μm. An additional step to this method could be an exfoliation through microwave or ultrasound, or both, thus obtaining thin layers GO [59].

Figure 7e,f shows the FESEM image corresponding to samples C1 and C2 (methodology C), respectively. The sample C1 irradiated with microwaves at 60 °C presented irregular laminar morphology, with high level of stacking layers, and leaf size between 1 and 3.96 μm; it is demonstrated that a complete degree of oxidation is obtained, based on the results of XRD and XPS.

In contrast, the C2 (Figure 7f) sample few layers was obtained with respect to the rest of the syntheses previously performed. However, this material presents a degree of oxidation similar to the cGO and C1, according to XRD, XPS, and Raman. Therefore, the expected result should be similar to the samples that are compared. Based on this result, this sample was analyzed by FETEM (JEOL, Akishima, Japan).

FESEM and FETEM analyzed the samples of methodology D. The sample D1 contains different rGO layers, with no apparent damage on the surface ascribed to the oxidation reaction observed in the micrograph shown in Figure 7g. Thus, few layers are observed in D2 (Figure 7h), which have great exfoliation and no damage on the surface. However, elemental analysis was performed with an energy dispersive system (EDS) with manganese content ~1% of debris or buildup, shown in Figure S9, which is difficult to remove when present in few layers rGO, according to what has been reported by other authors [60]. 

**Figure 8:**
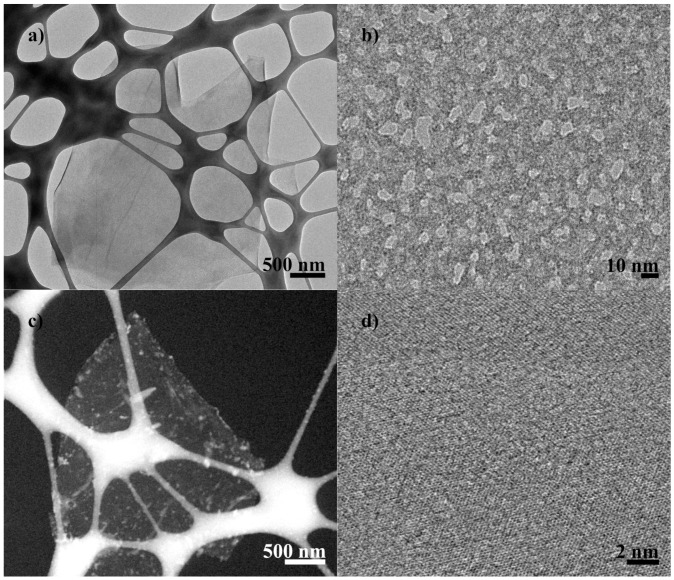
FETEM micrographs of high resolution mode STEM. (**a**,**b**) C2; (**c**,**d**) D2.

The samples C2 and D2 were studied using FETEM to analyze their morphology. C2 (see Figure 8a,b) presents highly inhomogeneous finding holes and high contrast disordered regions, indicating areas of high oxidation (Figure 8a), according to the results of XRD and XPS; establishing this presents increased exfoliation compared with cGO, as observed in the TEM micrograph of bright and dark field [46].

The FETEM image of sample D2 (see Figure 8c,d) exhibits few layers rGO observed as transparent sheets, but it presents debris or buildup of manganese or other salt products of oxidation processes. Figure 8d shows an FETEM micrograph in high resolution, in which large regions of defect-free graphene are clear.

## 3. Materials and Methods

### 3.1. Materials and Reagents

The GrO synthesis through the different methodologies was performed with a GG; previously, natural graphite flakes (GF, 99.99%, Alfa Aesar^®^, Haverhill, MA, USA) with a size of ≅10 mesh were ground in a Micro–Mill Scienceware^®^ (Bel-Art, Wayne, NJ, USA) to 10,000 rpm during 5 min. The reagents sodium nitrate (99%, Fermont, Monterrey, México), potassium permanganate (99.2%, Fermont, Monterrey, México) and sulfuric acid (96.2% *w*/*w* ACS grade by Fisher Scientific, Hampton, NH, USA) were used for the oxidation reaction of all methodologies at stage 1. Hydrogen peroxide (30%, Golden Bell, Anaheim, CA, USA) was employed in all methodologies, specifically at stage 3. The water tridestillated (Golden Bell, Anaheim, CA, USA) was used in different stages of the methodologies. The filters 541 Whatman™ (Sigma Aldrich, St. Louis, MO, USA) and Thermo Scientific membranes (4.5 μm, Waltham, MA, USA) were employed for purification of the carbon materials. Also, natural graphite powder (GN, 99.99%, Alfa Aesar^®^, Haverhill, MA, USA) was used for electrical conductivity measurements. 

### 3.2. Synthetic Procedures

#### 3.2.1. Method a Hummer’s Method

Stage 1: GG and sodium nitrate were mixed into a 250 mL three-necked flask in an ice bath with constant stirring. Then, sulfuric acid (46 mL) was induced by dripping; next, potassium permanganate was added slowly, with care taken to not exceed 20 °C. Mass ratio used for graphite, sodium nitrate, and potassium permanganate was 1:0.5:3, respectively. Five minutes later the three-necked flask was retired from the ice bath. Stage 2: The mixture was heated at temperature 35 ± 1 °C during 30 min. Subsequently, in the stage 3, tridistillated water (92 mL) was added gradually with constant stirring for 15 min. Stage 4: Hydrogen peroxide (80 mL) was added to the mixture with constant stirring until bubbles disappeared. Finally, in the stage 5, the product was purified through centrifuged rinse cycles, until the pH reached 5–6. The centrifugation speed was 4000 rpm for 15 min in each cycle. The product was filtered and dried in convection oven at 60 °C.

#### 3.2.2. Method B

Stage 1: GG and sodium nitrate were mixed into a 250 mL three-necked flask in an ice bath with constant stirring. Then, sulfuric acid (46 mL) was induced by dripping; next, potassium permanganate was added slowly, being careful to not exceed 20 °C. Mass ratio used for graphite, sodium nitrate, and potassium permanganate was 1:0.5:3, respectively. Five minutes later, the three-necked flask was retired from the ice bath. Stage 1b: The mixture is placed in quartz vials (50 mL), in which MWR (Multiwave PRO Anton Paar, Graz, Austria) was performed under the following conditions, see Table 1. Stage 2: The mixture was heated at temperature 35 ± 1 °C during 30 min. Subsequently, in the stage 3, tridistillated water (92 mL) was added gradually with a constant stirring during 15 min; in stage 4, hydrogen peroxide (80 mL) was added to the mixture with constant stirring until bubbles disappeared. Finally, in stage 5, the product was purified through centrifuged rinse cycles, until the pH reached 5–6. The centrifugation speed was 4000 rpm for 15 min in each cycle. The product was filtered and dried in convection oven at 60 °C.

#### 3.2.3. Method C

Stage 0: GG (2 g) was dispersed in distilled water (15 mL); it was set in a quartz vial and introduced in the microwave reactor (Multiwave PRO Anton Paar, Graz, Austria) at different temperatures under the conditions described in the Table 2. The GTMW was filtered, rinsed, and dried; later, it was introduced to a 250 mL three-necked flask. Stage 1: GTMW and sodium nitrate were mixed into a 250 mL three-necked flask in an ice bath with constant stirring. Then, sulfuric acid (46 mL) was induced by dripping; next, potassium permanganate was added slowly, with care taken not to exceed 20 °C. Mass ratio used for graphite, sodium nitrate, and potassium permanganate was 1:0.5:3, respectively. Five minutes later, the three-necked flask was retired from the ice bath. Stage 2: The mixture was heated at temperature 35 ± 1 °C for 30 min. Subsequently, in stage 3, tridistillated water (92 mL) was added gradually with constant stirring for 15 min. Stage 4; Hydrogen peroxide (80 mL) was added to the mixture with constant stirring until bubbles disappeared. Finally, in stage 5, the product was purified through centrifuged-rinse cycles, until the pH reached 5–6. The centrifugation speed was 4000 rpm for 15 min in each cycle. The product was filtered and dried in convection oven at 60 °C.

#### 3.2.4. Method D

Stage 0: GG (2 g) was dispersed in distilled water (15 mL); it was set in a quartz vial and introduced in the microwave reactor (Multiwave PRO Anton Paar, Graz, Austria) at different temperatures under the conditions described in Table 3. Stage 1: GG dispersed in water treated (GTMW) with MWR and sodium nitrate were mixed into a 250 mL three-necked flask in an ice bath with constant stirring. Then, sulfuric acid (46 mL) was induced by dripping; next, potassium permanganate was added slowly, with care taken to not exceed 20 °C. Mass ratio used for graphite, sodium nitrate, and potassium permanganate was 1:0.5:3, respectively. Five minutes later, the three-necked flask was retired from the ice bath. Stage 2: The mixture was heated at temperature 35 ± 1 °C during 30 min. Subsequently, in stage 3, tridistillated water (92 mL) was added gradually with constant stirring for 15 min. Stage 4: Hydrogen peroxide (80 mL) was added to the mixture at a constant stirring until bubbles disappeared. Finally, in stage 5, the product was purified through centrifuged rinse cycles, until the pH reached 5–6. The centrifugation speed was 4000 rpm for 15 min in each cycle. The product was filtered and dried in convection oven at 60 °C. Methodology D counterpart experiments were D2a and D2b, using untreated and treated water with MWR (60 °C, 20 min), respectively. In both cases GG without irradiation was used.

### 3.3. Characterization

XRD measurements of powders were analyzed on a D8 Advance (Bruker, Karlsruhe, Germany) with CuKα radiation (λ = 1.540598 Å). Data were collected from 4° to 40°, with a step size of 0.05° and a step time of 0.8 s. XPS of the different methods was conducted at a temperature of 25 °C on an ESCALAB 250 Xi (Thermo Scientific, Paisley, UK) using Kα excitation radiation (hm = 1486.6 eV). The pass energy was set at 4.2 eV. The angle employed was 45°, and the applied vacuum pressure was ~8–10 mbar. FTIR measurements of pristine graphite and samples were accomplished using a GX–FT–IR spectrometer (PerkinElmer, Waltham, MA, USA). Spectra were obtained by reflectance, employing a Total Attenuated Reflectance accessory. The samples were analyzed in transmittance mode in the range 600–4000 cm^−1^ with a resolution of 4 cm^−1^. Raman spectra of pristine graphite and samples were obtained using a LabRAM HR (HoribaJobin Yvon, Longjumeau, France) Vis–63 HeNe 632.8 nm laser. The Raman data acquisition was from 100 to 3500 cm^−1^ at room temperature. FESEM, JEOL JSM–7401F (JEOL, Akishima, Japan) and FETEM, JEOL JEM–2200FS (JEOL, Akishima, Japan) were used to investigate the surface morphology, size, and numbers of layers of GG and samples. The samples were dispersed in isopropyl alcohol and supported on a copper grid coated with a formvar/carbon 200 mesh film, letting them dry for a short period of time before being observed. The electrical conductivity and pH measurements were made using a PC18 digital pH-μS-°C-meter (Conductronic, Puebla, México).

## 4. Conclusions

The GrO synthesis with a different oxygen content was prepared through three modified versions of the Hummers method assisted MWR. Based on the methodologies, it can be established that it is possible to control the oxidation degree during reaction. Methodology D was an efficient and rapid process for the direct obtaining of rGO from GG in one-pot. The effectiveness of the methodology D could be of high importance for the preparation of the graphene materials. The use of MWR in the production of GO and rGO provides a novel method for the development of new graphene materials. The oxidation reaction has a synergistic effect between the dissociated water (mainly) and the delocalized π–electrons promoted by MWR. 

In all cases, the water treated with MWR plays an important role, because it decomposes the potassium permanganate and is enhanced when it is irradiated with microwaves, generating other species with different oxidation states to +7, suggesting a decrease in the oxidation reaction rate of the organic substrate. Additionally, the methodology D was conducted with a lower content of sulfuric acid compared with the Hummers method, obtaining rGO with low defects content. New approaches or strategies may emerge based on the results obtained from this research.

## Figures and Tables

**Table 1:** Reactions conditions used in method B (power 900 W and frequency 2455 MHz).

Sample	Temperature °C	Time (min)
B1	60	5
B2	60	20
B3	80	20

**Table 2:** Reactions conditions used in method C (power 900 W and frequency 2455 MHz).

Sample	Temperature °C	Time (min)
C1	60	5
C2	60	20

**Table 3:** Reactions conditions used in method D (power 900 W and frequency 2455 MHz).

Sample	Temperature °C	Time (min)
D1	60	5
D2	60	20

## Supplementary Materials

The following are available online at http://www.mdpi.com/2079-4991/8/2/106/s1, Figure S1: FESEM micrographs of ground graphite; Figure S2: Deconvolution of the X–ray diffraction patterns in the range 24–27° from samples B1, B2, B3 (methodology B) and the samples D1, D2 (methodology D); Figure S3: X–ray diffraction patterns of Graphite–stage 1 compared with B1, B2, B3; Figure S4: X–ray diffraction pattern of the ground graphite (GG), graphite treated with MW (GTMW), natural graphite (GN) and flakes graphite (GF); Figure S5: X–ray diffraction pattern from samples D2; D2b; D2a; GG; Figure S6: XPS survey spectrum of GG and GTMW; Figure S7: FTIR spectrum of graphites (GG, GTMW) and the samples D1, D2 (methodology D); Figure S8: Raman spectra deconvolution, band 2D, from samples B1, D1 and D2; Figure S9: (a) High–resolution TEM micrograph in STEM mode analyzing two areas of the sample D2. (b) EDS elemental analysis of the micrograph; Table S1: Results of the diffraction patterns and interplanar distance of all methods; Table S2: Water conductivity before and after microwave radiation; Table S3: Peak ratio intensities of *I_D_/I_G_* and *I*_2*D*_*/I_G_*.
