# Supplementary material for: Synthesis of Graphite Oxide with Different Surface Oxygen Contents Assisted Microwave Radiation
Source: Nanomaterials (Basel). 2018 Feb 13;8(2):106. doi: 10.3390/nano8020106 (PMC5853737; doi:10.3390/nano8020106)
Supplement: Supplementary file 1 [file nanomaterials-08-00106-s001.pdf]

Supplementary material

Synthesis of Graphite Oxide with Different Surface Oxygen Contents Assisted Microwave Radiation

Adriana Ibarra-Hernández [1], Alejandro Vega-Rios [1] and Velia Osuna [2,*]

1 Centro de Investigación en Materiales Avanzados, S.C., Miguel de Cervantes No. 120, Chihuahua 31136, Chihuahua., Mexico; adriibarra26@gmail.com (A.I.-H.); alejandro.vega@cimav.edu.mx (A.V.-R.)
2 Consejo Nacional de Ciencia y Tecnología (CONACYT)-Centro de Investigación en Materiales Avanzados, S.C., Miguel de Cervantes No. 120., Chihuahua 31136, Chihuahua., Mexico
* Correspondence: velia.osuna@cimav.edu.mx; Tel.: +52-614-439-4832

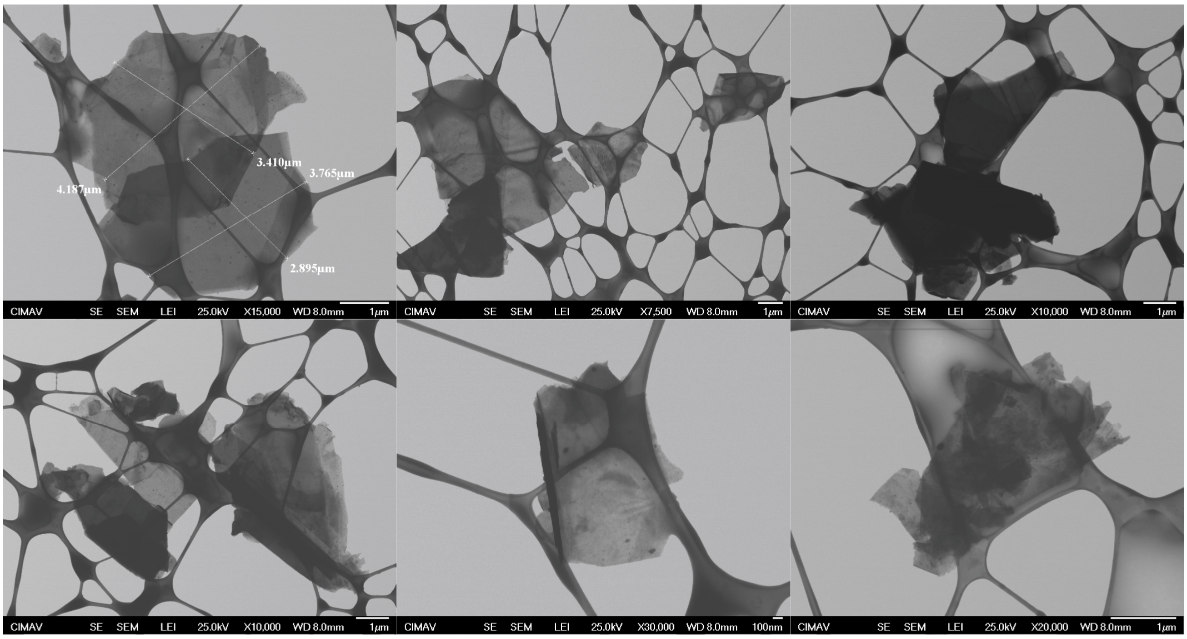

Figure S1. FESEM micrographs of ground graphite.

Table S1. Results of the diffraction patterns and interplanar distance of all methods.

Sample	Peak 1	d (A)	Peak 2*	d (A)	Peak 3*	d (A)	Peak 4	d (A)
**Method A**								
cGO	11.38	7.81	—	—	—	—	26.50	3.34
**Method B**								
B1	10.85	8.20	25.30	3.60	26.10	3.50	26.80	3.41
B2	11.80	7.53	25.50	3.57	25.90	3.53	26.40	3.46
B3	11.70	7.60	25.55	3.57	—	—	26.60	3.44
**Method C**								
C1	10.60	8.38	—	—	—	—	—	—
C2	10.60	8.38	—	—	—	—	—	—
**Method D**								
D1	10.4	8.53	—	—	—	—	—	—
D2	—	—	25.80	3.54	26.10	3.50	26.50	3.45

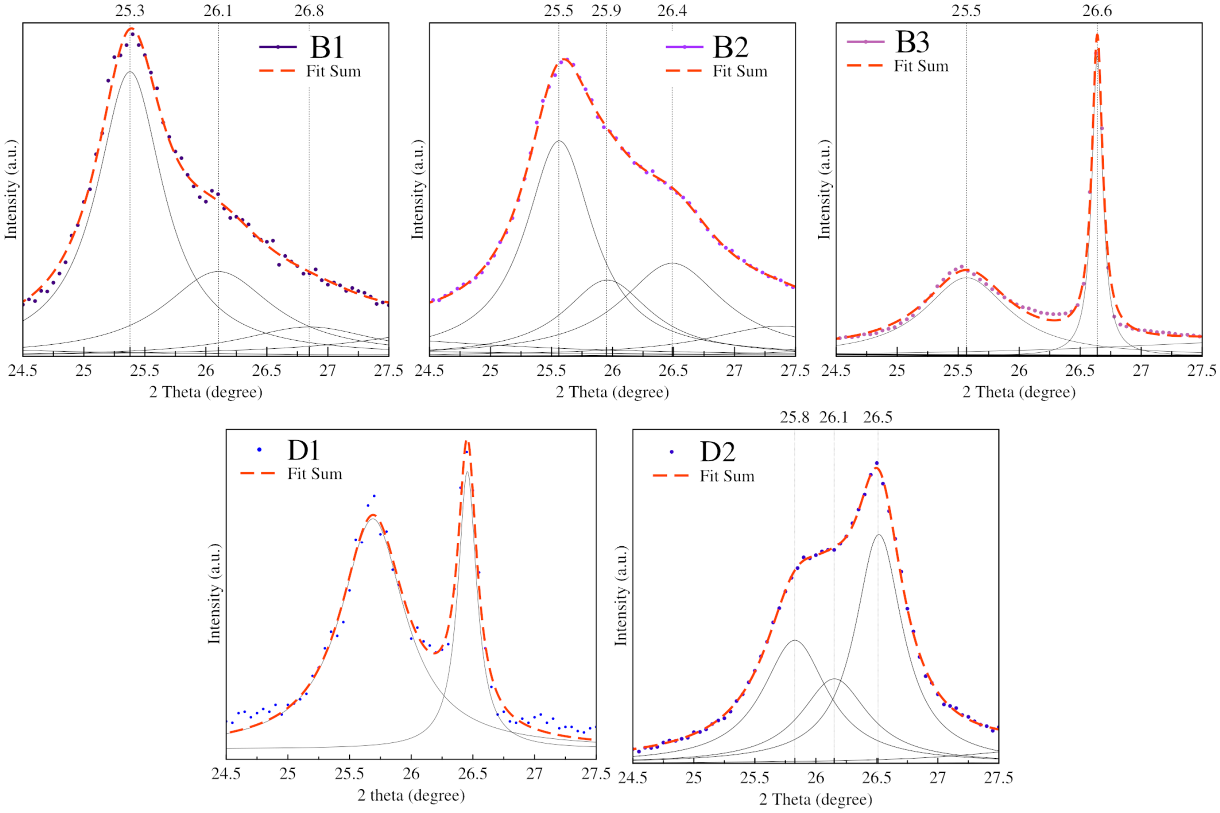

Figure S2. Deconvolution of the X–ray diffraction patterns in the range 24–27° from samples B1, B2, B3 (methodology B) and the samples D1, D2 (methodology D).

Deconvolution

The deconvolution of the diffractograms and Raman spectra were made using the software MagicPlot ver. 2.7.2 with 10000 interactions. Red line indicates a summation of curves deconvolution (Fit Sum), and solid black line represents the original experimental intensity from the background. Dotted black lines represent the assigned curves.

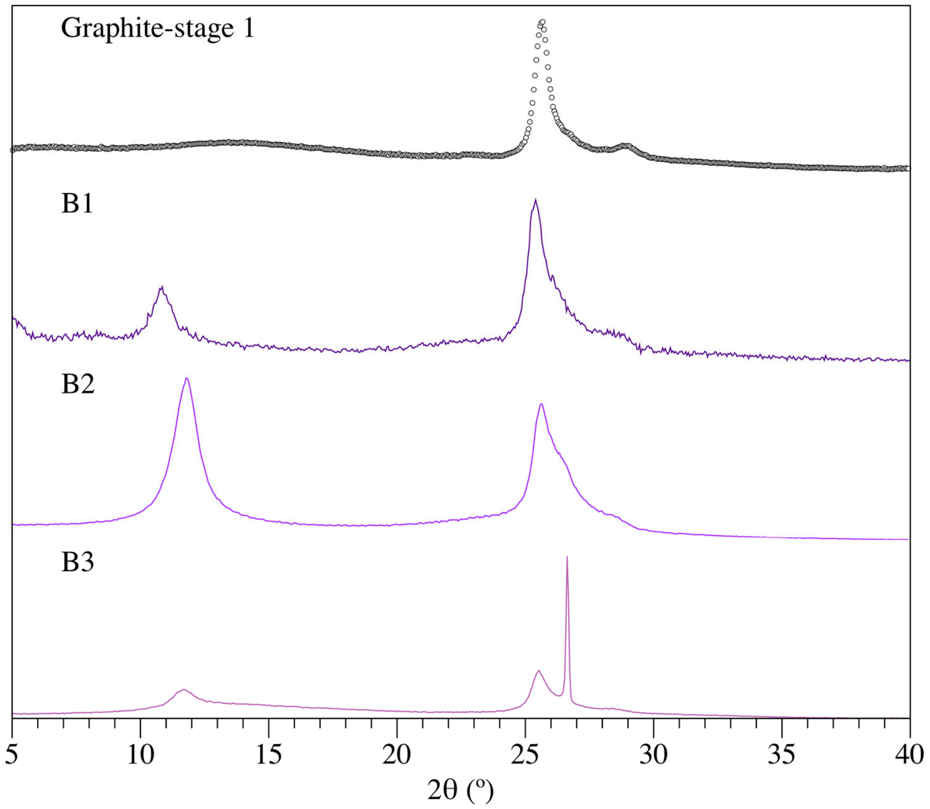

Figure S3. X–ray diffraction patterns of Graphite–stage 1 compared with B1, B2, B3.

Table S2. Water conductivity before and after microwave radiation*.

Sample	Description	Temperature (ºC)		Conductivity (μS)		pH	
		**Before**	**After**	**Before**	**After**	**Before**	**After**
Water	Water	17.5	17.6	9	13	7	7
GFT	Water + GF	22.1	22.0		57	7	7
GTMW	Water + GG		21.9		45	7	7
GNT	Water + GN		22.1		53	7	7

* The conditions of microwave radiation were at t=20 min and T=80ºC.

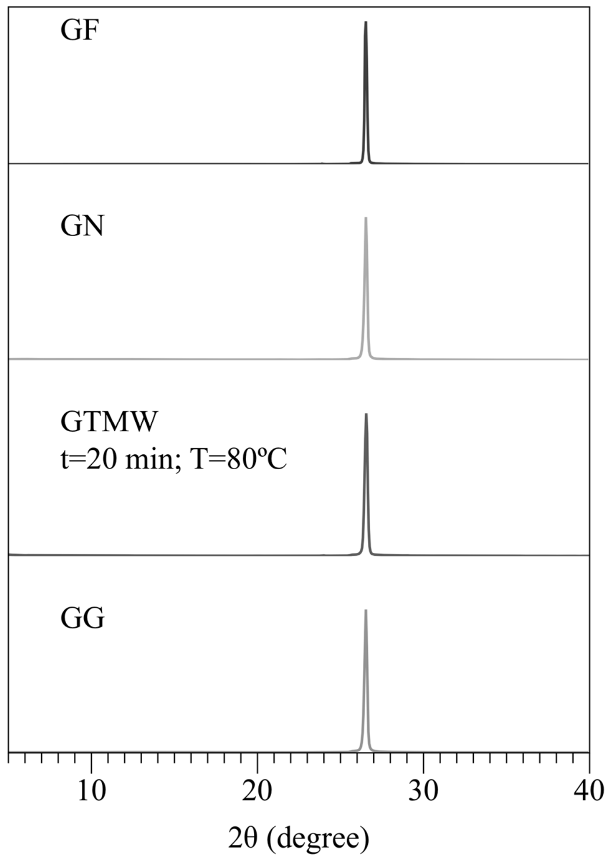

Figure S4. X–ray diffraction pattern of the ground graphite (GG), graphite treated with MW (GTMW), natural graphite (GN) and flakes graphite (GF).

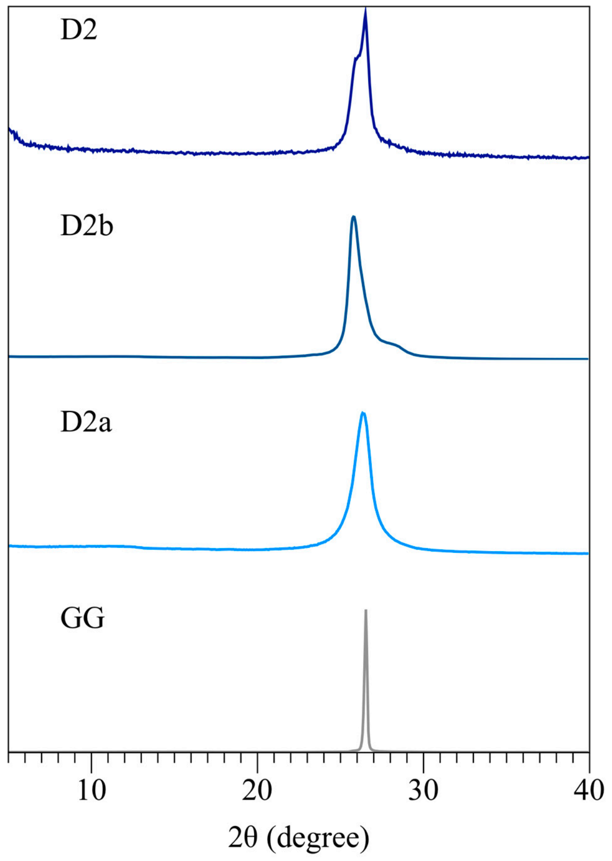

Figure S5. X–ray diffraction pattern from samples D2; D2b; D2a; GG.

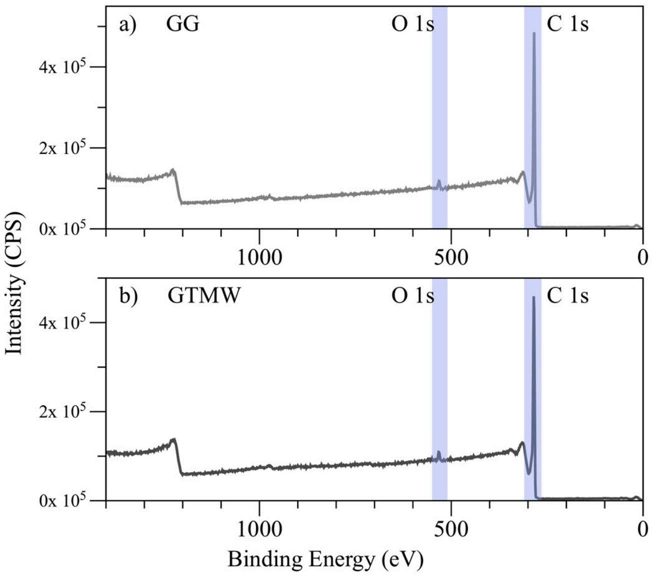

Figure S6. XPS survey spectrum of GG and GTMW.

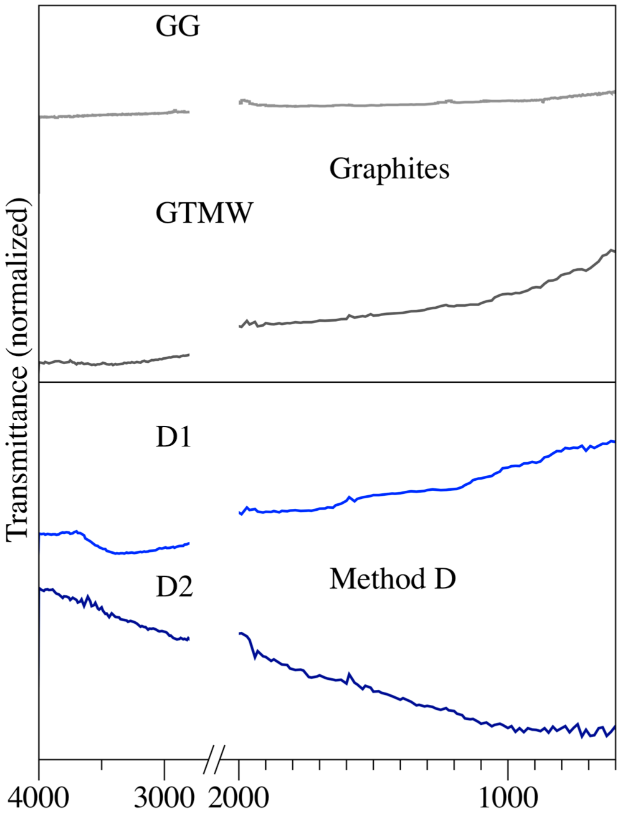

Figure S7. FTIR spectrum of graphites (GG, GTMW) and the samples D1, D2 (methodology D).

Table S3. Peak ratio intensities of I_D/I_G and I_{2D}/I_G.

**Sample**	I_D/I_G	I_{2D}/I_G
cGO	1.12	0.10
B1	0.79	0.34
B2	1.10	0.14
B3	0.99	0.22
C1	1.20	0.14
C2	1.07	0.13
D1	0.10	0.37
D2	0.20	0.42

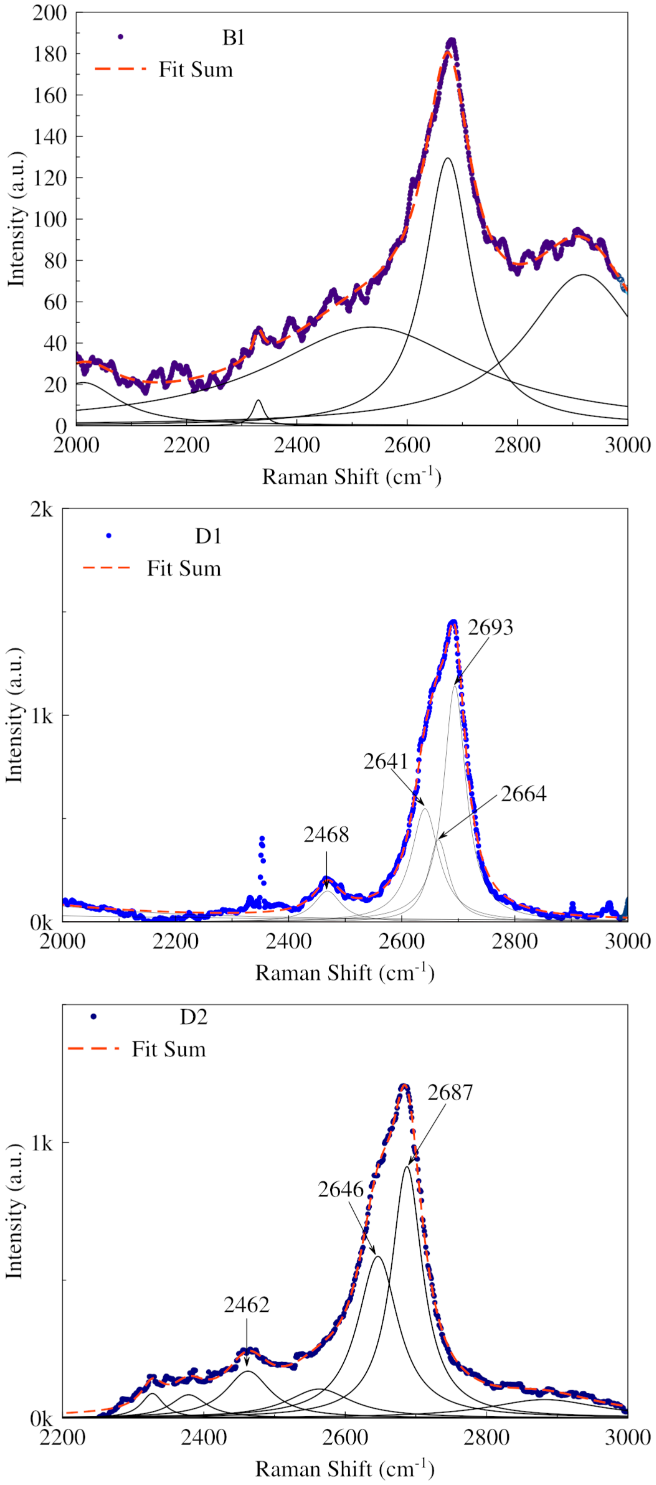

Figure S8. Raman spectra deconvolution, band 2D, from samples B1, D1 and D2.

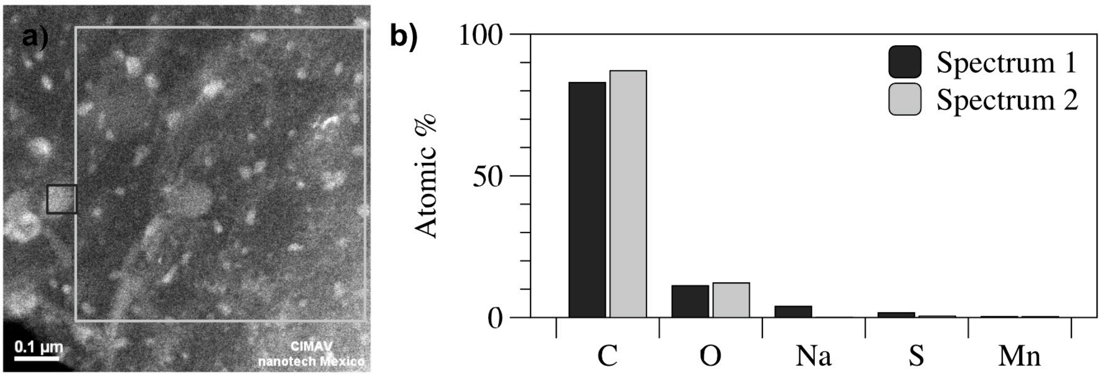

Figure S9. (**a**) High–resolution TEM micrograph in STEM mode analyzing two areas of the sample D2; (**b**) EDS elemental analysis of the micrograph.
